# Epigenome-wide DNA methylation and risk of breast cancer: a systematic review

**DOI:** 10.1186/s12885-020-07543-4

**Published:** 2020-10-31

**Authors:** Kaoutar Ennour-Idrissi, Dzevka Dragic, Francine Durocher, Caroline Diorio

**Affiliations:** 1 Department of Social and Preventive Medicine, Faculty of Medicine, Laval University, Quebec, QC Canada; 2 Laval University Cancer Research Center, Quebec, QC Canada; 3 Axe Oncologie, Centre de recherche du CHU de Québec-Université Laval, 1050 chemin Sainte-Foy, Quebec City, QC G1S 4L8 Canada; 4 Department of Molecular Biology, Medical Biochemistry and Pathology, Faculty of Medicine, Laval University, Quebec, QC Canada; 5 Department of Molecular Medicine, Faculty of Medicine, Laval University, Quebec, QC Canada; 6 Deschênes-Fabia Center for Breast Diseases, Saint-Sacrement Hospital, Quebec, QC Canada

**Keywords:** Breast cancer risk, DNA methylation, Epigenome-wide, HM450k, Systematic review

## Abstract

**Background:**

DNA methylation is a potential biomarker for early detection of breast cancer. However, robust evidence of a prospective relationship between DNA methylation patterns and breast cancer risk is still lacking. The objective of this study is to provide a systematic analysis of the findings of epigenome-wide DNA methylation studies on breast cancer risk, in light of their methodological strengths and weaknesses.

**Methods:**

We searched major databases (MEDLINE, EMBASE, Web of Science, CENTRAL) from inception up to 30th June 2019, for observational or intervention studies investigating the association between epigenome-wide DNA methylation (using the HM450k or EPIC BeadChip), measured in any type of human sample, and breast cancer risk. A pre-established protocol was drawn up following the Cochrane Reviews rigorous methodology. Study selection, data abstraction, and risk of bias assessment were performed by at least two investigators. A qualitative synthesis and systematic comparison of the strengths and weaknesses of studies was performed.

**Results:**

Overall, 20 studies using the HM450k BeadChip were included, 17 of which had measured blood-derived DNA methylation. There was a consistent trend toward an association of global blood-derived DNA hypomethylation and higher epigenetic age with higher risk of breast cancer. The strength of associations was modest for global hypomethylation and relatively weak for most of epigenetic age algorithms. Differences in length of follow-up periods may have influenced the ability to detect associations, as studies reporting follow-up periods shorter than 10 years were more likely to observe an association with global DNA methylation. Probe-wise differential methylation analyses identified between one and 806 differentially methylated CpGs positions in 10 studies. None of the identified differentially methylated sites overlapped between studies. Three studies used breast tissue DNA and suffered major methodological issues that precludes any conclusion. Overall risk of bias was critical mainly because of incomplete control of confounding. Important issues relative to data preprocessing could have limited the consistency of results.

**Conclusions:**

Global DNA methylation may be a short-term predictor of breast cancer risk. Further studies with rigorous methodology are needed to determine spatial distribution of DNA hypomethylation and identify differentially methylated sites associated with risk of breast cancer.

**Prospero registration number:**

CRD42020147244

**Supplementary Information:**

**Supplementary information** accompanies this paper at 10.1186/s12885-020-07543-4.

## Background

Alterations of DNA methylation patterns are the most common epigenetic aberrations in cancer and occur in cells during early breast cancer development and progression [1]. DNA methylation is a reversible biological signal that underlies tissue specific cell differentiation and cells adaptability to changes in their environment through regulation of gene expression [2]. Specifically, it is the addition of a methyl group to DNA cytosine bases that occurs predominantly in Cytosine-phosphate-Guanine (CpG) dinucleotides [2]. Approximately 60% of human genes contain high density of CpG dinucleotides in their promoters [3, 4]. CpG-rich regions are mostly unmethylated in normal cells when located in regulatory regions of housekeeping genes, tissue-specific genes and tumor suppressors [4, 5], while a methylated state of CpG islands located in promoters of some oncogenes leads to their transcriptional silencing [6].

As DNA methylation status of large subset of sites are known to be strongly correlated with each other, approaches that capture the dynamics of several sites simultaneously across the entire genome (epigenome-wide studies) are less prone to bias than candidate gene methylation studies [7]. Numerous genome-wide DNA methylation-profiling techniques exist, hindering the comparison of results across studies that have used different methods [8, 9]. While the whole-genome bisulphite sequencing method provides the highest accuracy and single nucleotide resolution, it is not yet feasible for large cohorts [9]. An acceptable compromise between coverage and precision is to target a comprehensive subset of the genome [9]. As such, the high-throughput and relatively affordable Infinium Human Methylation 450 K (HM450k) and MethylationEPIC (EPIC) BeadChip of Illumina, which targets approximately 480,000 CpG and 850,000 CpG sites across the human genome respectively, with at least 99% coverage of RefSeq genes [9, 10], have been widely used in epidemiological studies.

DNA methylation studies are aimed at identifying high-risk methylation patterns that may have an application in breast cancer early diagnosis and in identifying high-risk women for targeted interventions [11]. However, robust evidence of a prospective relationship between DNA methylation patterns and breast cancer risk is still lacking. Previous reviews focused mainly on whole-blood DNA methylation studies, considered all methods of DNA methylation measurement, the results of which are inherently different and difficult to compare across different methods, and lacked the systematic evaluation of strengths and weaknesses of included studies. Furthermore, many more epigenome-wide studies of breast cancer risk have been published since, prompting the need for an updated rigorous and systematic methodological evaluation of all relevant studies. Thus, the objective of the present systematic review is to evaluate and synthesize results of epigenome-wide association studies that have used the HM450k or EPIC BeadChip, to determine if global DNA methylation and specific differentially methylated sites are consistently associated with women breast cancer risk, and to identify what could have limited the consistency of their results.

## Methods

A systematic review was conducted following a pre-established protocol and the general methods for Cochrane reviews [12] and reported in adherence with PRISMA guidelines for systematic reviews and meta-analysis [13]. Considering the expected methodological diversity and heterogeneity between eligible studies, the great susceptibility of observational designs to selection bias and the variability in methods used to control for confounding, no quantitative synthesis was planned [12]. The protocol was deposited for registration at the International Prospective Register of Systematic Reviews (PROSPERO) in august 2019.

### Search methods for identification of studies

An electronic search was conducted in MEDLINE (via PubMed), EMBASE, Web of Science and CENTRAL (Cochrane Central Register of Controlled Trials) databases, from inception to June 30, 2019. Search strategies were developed for each of these databases with text words and index terms referring to breast cancer, methylation and risk (Table S1). No language or publication date restrictions were applied. The reference lists of relevant reviews as well as the included studies were scanned for any additional studies not otherwise identified.

### Criteria for considering studies for this review

#### Types of studies

Any observational or intervention study that evaluated the association between DNA methylation and breast cancer risk, whatever the study design, was eligible for inclusion. No restrictions were applied regarding language or publication type (articles, short reports and abstracts).

#### Types of participants

Women included in the studies before or after breast cancer diagnosis, regardless of age, stage, treatment regimen and menopausal status, were eligible. No participants were excluded based on ethnicity. A special attention was paid to identifying overlapping populations between studies, by comparing study population source, date of start and end of study recruitment, inclusion criteria, follow-up duration and population characteristics. When overlapping populations between studies was encountered, the study with the largest sample size was considered as the reference, and information was supplemented by the other publications as required.

#### Types of exposures

Only studies that measured DNA methylation in human samples (blood, breast tissue, breast fine needle aspiration, ductal fluid, human milk), on a genome-wide scale (epigenome-wide studies) using the HM450k or EPIC BeadChip were eligible. Measures of global DNA methylation across all included probes or a predefined set of probes (subset of CpGs defined by spacial localization or a pre-specified function such as epigenetic clocks algorithms) as well as probe-wise differential methylation analysis were considered appropriate exposure estimations.

#### Types of outcomes

Breast cancer risk, measured as breast cancer incidence, prevalence or breast mammographic density (a recognized breast cancer risk factor), or as defined by authors of included studies, was the primary outcome. Comparisons between matched normal and tumor tissue from the same patient were not considered a measure of breast cancer risk and were not included.

### Data collection and analysis

#### Selection of studies

The references identified by the search strategy were reviewed independently by two authors (KEI and CD) in a 2-step process. First, the title and abstract of each study were screened to exclude obviously non-eligible studies. Then, the full text of retained articles was examined and subjected to evaluation using the predefined eligibility criteria. Whenever required, a third review author (FD) was consulted. When required, further information was sought from the authors by email.

#### Data extraction

Data extraction was performed using an exhaustive standardized form designed for this review. Information about study design (inclusion criteria, sample size and methodology), participants and tumors characteristics at diagnosis (age, ethnicity, menopausal status, tumor invasiveness, tumor estrogen receptor (ER) status), exposure assessment (timing, tissue sample, tissue processing, data preprocessing methods), measured outcome and reported results (any reported measure of association, adjustment variables, and statistical model selection procedure) were collected. For observational studies, special attention was paid to distinguishing between adjusted and unadjusted results, and to the variable selection method used in multivariate analyses. The study’s definition of each retained characteristic or variable was recorded. In the case of multiple publications related to the same study, and to avoid the overlap across studies populations, the publication reporting the outcomes of interest to the present review or the one with the longest follow-up of these outcomes or with the largest sample size was considered as the reference, and information was supplemented by secondary publications as required. Abstracts with insufficient information and data to permit inclusion were excluded from the qualitative synthesis. Data were extracted independently by two review authors (KEI, DD) to ensure their consistency.

#### Assessment of risk of bias in included studies

Assessment of risk of bias was performed for each study and for the overall risk of bias across studies. Based on the “STrengthening the Reporting of OBservational studies in Epidemiology” (STROBE) statements [14], and the rating approach of the “Risk Of Bias in Non-randomized Studies - of Interventions” (ROBINS-I) tool [15], the following domains were evaluated for risk of bias in included studies: selection of participants into the study, exposure measurement, outcome measurement, potential confounding accounted for, missing data, and selective reporting. When required, a second reviewer (CD) was consulted.

#### Data synthesis

Given that high heterogeneity between studies was expected, quantitative synthesis of data was considered not appropriate. Using additional tables, a formal systematic qualitative and narrative synthesis of studies characteristics and results was performed separately for each type of tissue sample. A representative population sample was defined as one that includes at least 80% of postmenopausal patients and at least 80% of ER-positive invasive breast cancers [16]. The results were considered adjusted only when all important confounders were considered for adjustment. Authors should have considered age, body mass index or any other estimation of body fat, breastfeeding or parity, alcohol consumption and smoking as potential confounders. In addition, studies including multiple ethnic groups should have adjusted for ethnicity if no bioinformatics method was used to avoid population stratification bias. If authors, in the context of a particular study, demonstrated that a confounding factor is not associated to intervention or to outcome (i.e. a null association measure), and subsequently did not adjust for this factor, the results were considered adjusted [15]. In this context, a “no statistically significant association” was not considered a “no association” [15]. If authors have considered all important confounders for adjustment, and used an appropriate method for variable selection (i.e. backward selection method based on change in estimate) to reduce the number of adjustment covariates, the results were considered adjusted. A stepwise forward selection method or a selection method based on *p*-values were considered not appropriate as these methods are prone to introduce selection bias. A method based on change of estimate of odds ratio was considered inappropriate because of the non-collapsibility of such measures [17].

The direction and magnitude of observed associations across different statistical models were compared between studies for average methylation analyses, globally and by genomic regions. All individual differentially methylated CpGs identified from each study were compared to detect any overlapping CpGs. Results were considered consistent when associations were in the same direction across studies (at minimum in two studies) with no study reporting an opposite association. Any discrepancy was analyzed for sources of heterogeneity. A positive association was defined as an observed higher risk with higher methylation levels whereas a negative association was defined as an observed inverse association.

#### Assessment of heterogeneity

Differences between studies, including study design, participant characteristics (age and menopausal status), tumor characteristics (invasiveness, ER status and treatment received), exposure measurement (timing, type of tissue sample, preprocessing methods), statistical analysis (parametric or not, robust or not, adjusted or not) and different levels of risk of bias were considered to explore possible sources of heterogeneity.

## Results

### Results of the search

**Fig. 1:**
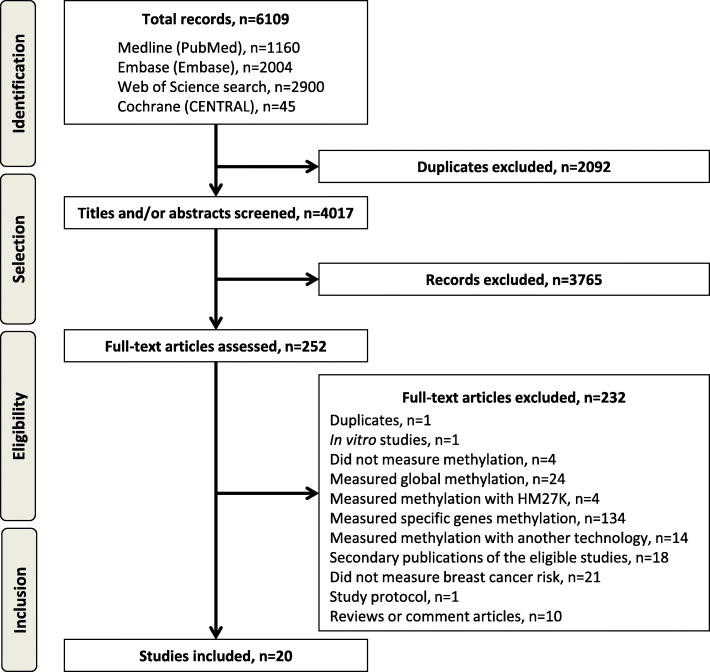
Flow Diagram according to PRISMA (Preferred Reporting Items of Systematic Reviews and Meta-Analyses) [13], with modifications

Of the 4017 references retrieved by electronic search after duplicate removal, 20 studies, published between 2013 and 2019, met eligibility criteria (Fig. 1) [13], of which 17 measured blood-derived DNA methylation [18–34] and three measured breast tissue DNA methylation [35–37].

### Description of studies

#### Studies of blood-derived DNA methylation

Characteristics of the 17 studies of blood-derived DNA methylation are reported in Table 1 and Table S2. These studies involved between 90 and 228,951 participants (median = 465 participants), including 48 to 122,977 cases (median = 233 cases) drawn from one to four different populations. Most studies were nested case-control studies, were conducted on populations from European countries, spanned from 2 weeks to over 20 years of follow-up and evaluated incident breast cancer risk. All studies used the HM450k beadchip. One study aggregated methylation data of common CpGs retrieved from four different populations, of which one have used the EPIC beadchip [19].

**Table 1:** Summary characteristics of blood-derived methylation studies and breast cancer risk (*n* = 17)

**Design**	*Study design* Case-cohort or cohort studies, *n* = 2 Nested case-control studies, *n* = 9 Unspecified case-control studies, *n* = 3 Cross-sectional study, *n* = 1 Multiple designs, *n* = 2 *Sample size* Total participants, 90 to 228,951 Breast cancer patients, 48 to 122,977	*Population source* Europe, *n* = 8 Australia, *n* = 3 USA, *n* = 2 Europe and/or Australia and/or USA, *n* = 4 *Follow-up* Duration, 2 weeks to > 20 years Not reported in 9 studies
**Breast cancer patients**	Mean age, 48 to 64 years old Postmenopausal, 31 to 100%, NR in 10 studies	Invasive cancers, 88 to 100%, NR in 10 studies ER-positive cancers, 0 to 83%, NR in 9 studies
**DNA methylation measurement**	*Timing* Before diagnosis, *n* = 13 After diagnosis, before treatment, *n* = 2 After diagnosis, unspecified, *n* = 1 Not reported, *n* = 1 *Cell-type proportions* Estimated (Houseman algorithm), *n* = 10 Estimated, other method, *n* = 2 Estimated, method NR, *n* = 2 Not considered, *n* = 3 *Probe design bias correction method* Functional normalization^a^, *n* = 7 SWAN^a^, *n* = 7 BMIQ, *n* = 2 Quantile normalization, *n* = 2 RCP, *n* = 1 Not reported, *n* = 4	*Cross-hybridizing probes* Excluded, *n* = 5 Not reported, *n* = 12 *Probes with SNP* Excluded, *n* = 5 Not excluded, *n* = 1 Not reported, *n* = 11 *X chromosomes* Excluded, *n* = 5 Included, *n* = 4 Not reported, *n* = 8
**Outcomes**	Breast cancer incidence, *n* = 16 Breast mammographic density, *n* = 1	
**Statistical modeling**	**Global methylation,** ***n*** **= 9** *Type of global methylation analysis* Average across all included probes^c^, *n* = 6 Average across pre-defined set of probes^c^, *n* = 5 *Type of methylation value* Beta-values, *n* = 8 Not reported, *n* = 1 *Statistical model* Logistic regression, *n* = 5 Cox proportional hazard model, *n* = 1 Non-parametric test, *n* = 2 Not reported, *n* = 1 *Adjustment* Appropriate, *n* = 3 Incomplete, *n* = 4 None, *n* = 2	**Probe-wise differential methylation,** ***n*** **= 16** *Type of methylation value* Beta-values, *n* = 10 M-values, *n* = 4 Not reported, *n* = 2 *Statistical model* Logistic regression^b^, *n* = 6 Cox proportional hazard models^b^, *n* = 2 Beta-regression, *n* = 2 Linear mixed effect model, *n* = 2 MetaXcan method, *n* = 1 Linear regression with empirical Bayes methods, *n* = 1 Non-parametric tests, *n* = 1 Not reported, *n* = 2 *Adjustment* Appropriate, *n* = 3 Incomplete, *n* = 12 None, *n* = 1 *Multiple comparison correction* Bonferroni’s correction, *n* = 6 FDR, *n* = 3 None, *n* = 7

*n* number of studies, *NR* not reported, *SNP* single nucleotide polymorphism, *SWAN* Subset-quantile within array normalization, *BMIQ* Beta-mixture quantile normalization, *RCP* Regression on Correlated Probes, *DMP* differentially methylated positions, *FDR* false discovery rate, *ER* estrogen receptor

^a^*n* = 6 studies used both functional normalization and SWAN

^b^one study used both logistic regression and Cox proportional hazard models

^c^*n* = 2 studies measured both

Studies included breast cancer patients between 48 and 64 years of mean age (*n* = 12 studies), and one study included exclusively patients under 40 years old [28]. Proportion of postmenopausal patients varied from 31 to 100% (*n* = 7 studies), with only one study including at least 80% of postmenopausal patients [26]. Proportion of patients presenting invasive breast cancers varied from 88 to 100% (*n* = 7 studies), with five studies including exclusively invasive breast cancers [18, 19, 21, 30, 31]. Proportion of ER-positive breast cancers varied from 0 to 83% (*n* = 7 studies), with only two studies including at least 80% of ER-positive breast cancers [30, 32], and one study including exclusively ER-negative breast cancers [22] (Table 1 and Table S2).

Most studies measured blood-derived DNA methylation in samples collected before cancer diagnosis, estimated blood cell-type proportions using Houseman’s algorithm and used functional normalization to correct for probe design bias. Few studies mentioned exclusion of cross-hybridizing probes, probes containing SNPs, and probes located on X-chromosomes (Table 1 and Table S2). Most studies reporting global methylation analysis across all included probes or a predefined set of probes used methylation beta-values in conditional or unconditional logistic regression models, with only three out of nine studies considering all important confounders for adjustment [23, 24, 27]. Most studies reporting probe-wise differential methylation analysis used methylation beta-values, conditional or unconditional logistic regression models, Bonferroni’s correction for multiple comparisons, with only three out of 16 studies considering all important confounders for adjustment [23, 24, 27] (Table 1 and Table S2).

#### Studies of breast tissue DNA methylation

Characteristics of the three studies of breast tissue DNA methylation are reported in Table S3 [35–37]. These studies involved between 96 and 262 participants, including 35 to 210 cases, drawn from hospital and tissue bank registries, with one study using The Cancer Genome Atlas (TCGA) data [35]. These studies were mainly cross-sectional and used samples collected after breast cancer diagnosis, with only one study reporting samples collection before any treatment [36]. All three studies used the HM450k beadchip [35–37].

Included patients in these studies were between 50 and 61 years of mean age, with one study including 29% of patients under 49 years old [37]. One study included 33% of postmenopausal patients [36], and two studies included more than 80% of invasive breast cancers [35, 37]. Proportions of ER-positive breast cancers varied from 63 to 98%, with two studies including more than 80% of ER-positive tumors [35, 36] (Table S3).

Only one study compared normal breast tissue of cases to normal breast tissue of non-cases [36], whereas one study compared tumor tissue of cases to normal tissue of non-cases [37] and the third one compared tumor tissue collected from breast cancer patients to normal breast tissue collected from a different group of breast cancer patients [35]. No study verified cell composition of collected samples nor considered correction for cell-type proportions. Correction for probe design bias was reported by two studies [36, 37], whereas only one study mentioned exclusion of cross-hybridizing probes and probes containing SNPs [37] and none of them reported exclusion of probes located on sex chromosomes. All three studies used methylation beta-values, nonparametric tests for global methylation and probe-wise differential methylation analyses, and Benjamini-Hochberg’s correction for multiple comparisons. No study performed appropriate adjustment for breast cancer risk and prognostic factors to control for confounding and reverse causation bias.

### Risk of bias in included studies

Overall, studies ranged from moderate to serious risk of bias, with most studies reporting insufficient information on selection of participants into the study and handling of missing data to enable evaluation of risk of selection bias. Most studies that included multiple ethnic groups did not investigate nor correct for population stratification bias, and few studies controlled appropriately for potential confounding factors.

### Systematic data synthesis

#### Studies of blood-derived DNA methylation

Among the nine studies reporting global methylation analysis, six measured average methylation across all included probes, of which one estimated separate associations in three different populations [33] (Table S2). Out of the eight separate association analyses, four identified a global hypomethylation in women who developed breast cancer, with odds ratios ranging from 0.69 [0.50–0.95] [31, 38] to 0.94 [0.85–1.05] [19], and one study reported a trend toward a marginally lower average methylation in breast cancer patients [32] (Table S2). The three other analyses did not identify a difference between cases and controls [25, 29, 30], and no study reported an opposite association. Three studies reported analyses by CpG location [30, 33, 38], of which one reported higher CpGs islands methylation in breast cancer patients [30] whereas the two other studies did not identify an association with breast cancer risk (Table S2). One study also reported higher methylation in CpGs located in functional promoters but lower methylation in CpGs located far from islands and CpGs located outside promoters in association with breast cancer risk [38].

Five studies measured average methylation across a pre-defined set of probes, four of which corresponded to estimations of epigenetic age using different published algorithms such as Horvath (353 CpGs, *n* = 4 studies) [25, 29, 30, 36], Hannum (71 CpGs, *n* = 3 studies) [25, 29, 36], Levine (513 CpGs, *n* = 1 study) [25] and Weidner (3 CpGs, *n* = 1 study) [29] epigenetic clocks. Higher Horvath’s epigenetic age was associated with 4 to 9% higher risk of breast cancer in three out of four studies (OR = 1.04 [1.01–1.08] [30], HR = 1.08 [1.00–1.17] [25], and HR = 1.09, *p*-value 6.3 × 10^− 5^ [36]), with one study reporting no association [29]. One study reported 10% higher risk of breast cancer with higher Hannum’s epigenetic age (HR = 1.10 [1.00–1.21]) [25], whereas the two other studies reported no association [29, 36]. One study reported 15% higher risk of breast cancer with higher Levine’s epigenetic age (HR = 1.15 [1.07–1.23]) [25] whereas the study that estimated Weidner’s epigenetic age reported no association with breast cancer risk [29]. One study calculated a methylation index based on 31 CpGs associated with estimated lifetime estrogen exposure and reported 43% higher breast cancer risk in the fourth vs first quartile of methylation index (OR = 1.43 [1.05–2.00]) [18].

Sixteen studies performed probe-wise differential methylation analyses, of which one study performed separate association analyses in two different populations [33]. Out of the 17 probe-wise differential methylation analyses, seven did not identify associations with breast cancer risk, of which one study reported two differentially methylated CpGs positions (DMP) when restricting analyses to cases occurring within 2 years of blood draw [19]. The other 10 probe-wise differential methylation analyses identified between one and 806 DMP (median = 24 DMP) with no overlapping DMP between different studies. Five genes overlapped between two different studies but differed in the identified DMP, namely: *GRB10* [18, 25], *RPH3AL* [18, 25], *SEMA5A* [25, 33], *C7orf50* [25, 27] and *XYLT1* [22, 27].

#### Studies of breast tissue DNA methylation

The one study that measured average methylation across all included probes reported higher methylation in tumor tissue of cases than in normal tissue of controls [37], globally and in CpGs located in islands and shores whereas CpGs located in shelves and “open sea” were hypomethylated in tumor tissue of cases [37]. One study measured average methylation across a predefined set of probes corresponding to Horvath’s clock and reported higher epigenetic age in normal breast tissue of cases when compared with normal breast tissue of controls [36] (Table S3).

Two studies performed probe-wise differential methylation analyses and reported respectively 550 [35] and 2761 DMP [37] between tumor and normal tissue. Detailed analysis of overlapping DMP was not performed because the list of DMP was not reported in one study.

No overlapping DMP was identified between studies of blood-derived DNA methylation and studies of breast tissue DNA methylation. Thirteen genes (*IGF2BP, HIST1H3E, CUBN, ADCY4, ZNF804A, HIST1H1A, NOX4, CYP24A1, GLIPR1L1, CHODL, PLSCR4, CDH26* and *RAD54B*) overlapped between a study of blood-derived DNA methylation [25] and the study of tumor vs normal breast tissue of different breast cancer patients [35] but differed in the identified DMP.

#### Assessment of heterogeneity

Overall, patients age was not related to the observed differences between studies results. Insufficient information was available to evaluate the impact of other population characteristics, such as menopausal status, and tumor characteristics, such as tumor invasiveness and ER status. Studies that have identified an association between global methylation and breast cancer risk reported follow-up periods shorter than 10 years, and one study reported stronger associations after restricting analyses to the first 5 years to 10 years after blood draw [38]. However, this observation was not reflected by differences in time to diagnosis for cases and was not evaluated in studies reporting probe-wise differential methylation analyses because of lacking information.

## Discussion

The present systematic review of epigenome-wide DNA methylation and risk of breast cancer indicates a consistent trend toward a global blood-derived DNA hypomethylation and higher estimates of epigenetic age in women who develop breast cancer. None of the identified differentially methylated CpGs in individual studies were consistently associated with breast cancer risk across studies and sparse data precludes any conclusions from studies of breast tissue DNA methylation.

Although the overall strength of evidence is weak, since most studies were at least at serious risk of bias and the strength of associations is relatively weak, especially for epigenetic age, our findings are more consistent than those observed from studies that have used other global DNA methylation estimation methods such the luminometric methylation assay (LUMA), liquid chromatography-mass spectrometry (LC-MS) of 5-methyldeoxycytosine (5-mdC) concentration or pyrosequencing and MethyLight assay measuring the methylation of repetitive DNA elements (i.e., LINE-1, Alu, or Sat2) [39], indicating that these methods may not capture the global DNA methylation differences between cases and controls.

A growing body of evidence suggests that well known breast cancer risk factors are associated with global DNA hypomethylation and increased epigenetic age [40], including lifestyle and dietary factors [41, 42], body mass index [43], physical inactivity [44], and hormone exposure [45]. Furthermore, global DNA hypomethylation has been observed in cancers [46], including breast carcinomas, indicating that DNA methylation mediates gene-environment interactions. However, effect of DNA hypomethylation depends on the genomic location of hypomethylated CpGs [47]. In fact, while DNA hypomethylation of gene promoters is positively correlated with gene transcription, hypomethylation in repetitive elements may lead to genomic instability and reactivation of expression of transposable elements, whereas hypomethylation within gene bodies may disturb alternative splicing [47]. Even though few studies included in the present systematic review have considered CpGs location in their analyses, there is some indication that the variability in DNA methylation between breast cancer cases and controls is driven by differential methylation of CpGs located outside CpGs islands and promoters.

The lack of evidence for consistent associations between DNA methylation at specific CpGs and breast cancer risk may be explained by methodological biases. Because DNA methylation profiles, unlike the genome, are subject to dynamic changes induced by genetic, environmental and stochastic factors [9], identification of a causal relationship is challenging and requires the use of conventional epidemiological approaches [9], which has been largely overlooked in most included studies.

In addition to traditional causes of biases inherent in observational designs, an important issue was related to preprocessing of methylation data. Different methods for data normalization have been developed for probe design bias correction, a systematic difference in methylation values distributions related to the use of two types of probes of different chemical properties in the HM450k BeadChip. While no single normalization method is considered the best, functional normalization method, which was used by most included studies, is appropriate for cancer/normal comparisons and vastly different tissue types, where large global methylation differences are expected [48]. When comparing the same tissue type, functional normalization method is believed to be inappropriate as it may obscure true differences between individuals [48]. Moreover, few studies reported exclusion of cross-hybridizing probes and probes overlapping SNPs prior to analyses, which are known to generate technical and biological artifacts that could have confounded the results [49].

The strengths of the present systematic review include the use of the Cochrane Reviews rigorous methodology, the extensive and highly sensitive search strategy to retrieve as many relevant studies as possible, the use of a pre-established protocol, the assessment of the risk of bias, and the systematic analysis of results in light of methodological strengths and weaknesses of relevant studies. Limitations include the lack of high-quality evidence and the overall serious risk of bias in included studies, due to selection bias, confounding and data preprocessing.

Although considered relatively stable, DNA methylation is a labile and reversible feature that may vary over time, reflecting variation in environmental exposures [50]. In fact, we observed that differences in follow-up periods may have impacted detection of differences in methylation patterns between breast cancer cases and controls, suggesting that a point measurement of DNA methylation may not predict lifetime breast cancer risk, but rather could be used for short-term prediction of breast cancer risk. It should also be kept in mind that DNA methylation patterns are tissue-specific. While tissue-specificity is generally considered of lesser concern in studies aiming at identification of biomarkers of exposure or disease risk, DNA methylation patterns obtained from accessible surrogate tissues such as blood can not be easily extrapolated to breast tissue [11]. In fact, concordance between DNA methylation in different tissues seems to be complex and locus dependent [51] and if high inter-tissue correlation may be present when methylation changes induced during embryogenesis are propagated soma-wide, changes occurring during adulthood and ageing are more likely to remain tissue specific [9, 51, 52]. For DNA methylation biomarkers to have the potential to inform interventions based on epigenetic agents for prevention or treatment of breast cancer, it is necessary to demonstrate a mechanistic link between DNA methylation patterns and breast cancer occurrence [11]. Such mechanistic link could only be supported by identification of tissue-specific DNA methylation changes in normal breast tissue prior to breast cancer occurrence [11].

To overcame some of the observed limitations, epigenome-wide studies should use more conventional epidemiological approaches, including an ethnically homogeneous and representative sampling of breast cancer patients and proper selection of controls to minimize the risk of selection bias (such as the use of nested case-control designs). Moreover, appropriate correction of potential confounding (by adjusting or matching for breast cancer known risk factors) should be considered. Studies should also allow for a sufficient lag time (time between sample collection and breast cancer diagnosis) to minimize the risk of reverse causation (effects of an underlying breast cancer not yet diagnosed). In addition, studies should consider the impact of time to diagnosis for cases and length of follow-up in controls as changes in methylation status due to variation in environmental exposures can occur during long follow-up periods and bias the observations toward the null (toward weaker associations or no association). Finally, data preprocessing should avoid functional normalization methods, which are not suitable for detection of discreet differences between samples from the same tissue type, and should exclude cross-hybridizing probes and probes overlapping SNPs prior to analyses.

While epigenome-wide DNA methylation methods are particularly suitable for hypothesis generation, as they capture the dynamics of several sites simultaneously across the entire genome, their findings, particularly differential methylation of specific CpGs sites and related genes, should be validated using a different measurement method, with higher sensitivity and specificity, such as PCR-based methods in a candidate-gene methylation approach. In addition, any detected methylation differences should be supplemented by transcriptional or protein expression analysis to confirm their functional impact and its association with breast cancer occurrence [53]. Once validated, specific CpGs methylation status, and expression value of related genes, could be used in prospective study designs to generate comprehensive predictive models, integrating clinical characteristics and environmental risk factors that would accurately predict breast cancer risk for each woman.

## Conclusions

Since the launch of the high-throughput HM450k BeadChip for epigenome-wide interrogation of DNA methylation, many epigenome-wide studies have tried to identify high-risk methylation patterns associated with breast cancer risk. Despite methodological differences between studies, we observed a trend toward an association of global blood-derived DNA hypomethylation and higher epigenetic age with breast cancer risk in women. Further epigenome-wide studies should use more conventional epidemiological approaches, including an ethnically homogeneous and representative sampling of breast cancer patients, proper selection of controls and proper correction of potential confounding, in addition to considering the impact of time to diagnosis for cases and length of follow-up in controls and choosing proper data preprocessing methods.

## Supplementary Information

**Additional file 1 **: **Table S1.** Search strategy for Medline via PubMed. **Table S2.** Studies of blood-derived methylation and breast cancer risk. **Table S3.** Studies of breast tissue methylation and breast cancer risk.

## Data Availability

The dataset(s) supporting the conclusions of this article are included within the article and its additional file.

## Abbreviations

DNA: Deoxyribonucleic acid
CpG: Cytosine-phosphate-Guanine
HM450k: Human Methylation 450 k
ER: Estrogen receptor
HR: Hazard ratio
DMP: Differentially methylated CpGs positions
FFPE: Formalin fixed paraffin embedded
