# Supplementary material for: Epigenome-wide DNA methylation and risk of breast cancer: a systematic review
Source: BMC Cancer. 2020 Oct 31;20:1048. doi: 10.1186/s12885-020-07543-4 (PMC7603741; doi:10.1186/s12885-020-07543-4)
Supplement: Supplementary file 1 — Additional file 1 : Table S1. Search strategy for Medline via PubMed. Table S2. Studies of blood-derived methylation and breast cancer risk. Table S3. Studies of breast tissue methylation and breast cancer risk. [file 12885_2020_7543_MOESM1_ESM.docx]

Table S1. Search strategy for Medline via PubMed

Table S2. Studies of blood-derived methylation and breast cancer risk

Table S3. Studies of breast tissue methylation and breast cancer risk

| **Table S1. Search strategy for Medline via PubMed** |
| --- |
| (("Breast"[tiab] OR "mammary gland"[tiab] OR "mammary" [tiab]) AND ("cancer" [tiab] OR "cancers" [tiab] OR "carcinoma" [tiab] OR "carcinomas" [tiab] OR "malignant neoplasm"[tiab] OR "malignant neoplasms"[tiab] OR "neoplasm"[tiab] OR "neoplasms"[tiab] OR "malignant tumor"[tiab] OR "malignant tumors"[tiab] OR "malignant tumour"[tiab] OR "malignant tumours"[tiab] OR "tumor"[tiab] OR "tumors"[tiab] OR "tumour"[tiab] OR "tumours"[tiab] OR "malignancy"[tiab] OR "malignancies"[tiab])) OR "Breast Neoplasms"[Mesh] |
| ***AND***  "Methylation" [tiab] OR "Methylations" [tiab] OR "Epigenetic" [tiab] OR "Epigenetics" [tiab] OR "Epigenomic"[tiab] OR "Epigenomics"[tiab] OR "Epigenesis" [tiab] OR "Epigenome" [tiab] OR "Epigenomes" [tiab] OR "Methylome"[tiab] OR "Methylomes"[tiab] OR "Hypermethylation"[tiab] OR "Hyper-methylation"[tiab] OR "Hyper methylation"[tiab] OR "Hyper methylations"[tiab] OR "Hypermethylations"[tiab] OR "Hyper-methylations"[tiab] OR "Hypomethylation"[tiab] OR "Hypo-methylation"[tiab] OR "Hypo methylation"[tiab] OR "Hypo methylations"[tiab] OR "Hypomethylations"[tiab] OR "Hypo-methylations"[tiab] OR "Methylation"[Mesh] OR "Epigenomics"[Mesh] |
| ***AND***  "Risk"[tiab] OR "Risks"[tiab] OR "Incidence"[tiab] OR "Incidences"[tiab] OR "occurrence"[tiab] OR "occurrences"[tiab] OR "prevalence"[tiab] OR "prevalences"[tiab] OR "Risk"[Mesh] OR "Incidence"[Mesh] |
| ***NOT*** (animals [mh] NOT humans [mh]) |

| **Table S2. Studies of blood-derived methylation and breast cancer risk** | | | | | | | | | | |
| --- | --- | --- | --- | --- | --- | --- | --- | --- | --- | --- |
| **Study reference(s)** | **Population**  **source** | **Design**  Mean (SD), [range] | **Patients and tumors characteristics***  Mean (SD), [range] | **Methylation measurement** | **Preprocessing and statistical modeling** | **Statistical modeling** | | **Reported associations** | | **Comments** |
|  |  |  |  |  |  | **Model(s)** | **Adjustment** | **Average** | **DMP** |  |
| Johansson A. et al. 2019 [1] | . EPIC-Italy  . Generations  Study (UK)  . EPIC-IARC  (Europe)  . MCCS  (Australia) | . Nested case-control study  . Lag-time: not considered  . Total n: 678 to 2374  . Cases n: 339 to 1187  . Follow-up: NR  *mean time to diagnosis* *from 4.0 (2.4) to 7.9 (3.8) years* | . Age: 51.3 (7.3) to 56.7 (8.2) years  . Post-menopausal: 48.3 to 60.2%  . Invasive: 100%  . ER+: NR | . Timing: before cancer  . Time to diagnosis: NR  . Dried blood spots, buffy coat, or lymphocytes  . Cell-type proportions: Houseman estimation  . CpGs: methylation index based on 31 CpGs | . Functional normalization (Illumina) and SWAN  . Detection p-value <0.01  . Cross-hybridizing probes: excluded  . SNP probes: NR  . Y chrom: excluded  . X chrom: included | . Beta-values  . Beta-regression for association with ELEE then conditional logistic regression  . p<0.05  *(ELEE: estimated lifetime estrogen exposure)* | *Selection method:* NR  *Variables:*  . Age  . BMI  . Alcohol consumption  . Smoking duration  . Cell-type proportions  *(Batch and position on batch for ELEE model)* | *Methylation index, Q4 vs Q1:*  OR=1.43  [1.05-2.00] | 4 CpGs  cg06968859  cg08835688  cg22758104  cg19216791  *(n=162 pairs)* | . 42/694 CpG probes associated with ELEE (FDR <0.05) selected for target sequencing (∆>0.1%, P< 7×10^−5^) of which 31 CpG passed QC  . Incomplete adjustment for breast cancer risk factors |
| Bodelon C. et al. 2019 [2] | . MCCS  (Australia)  . EPIC-Italy  . EPIC-IARC  (Europe)  . PLCO (USA) | . Nested case-control study  . Lag-time: not considered  . Total n: 3548  . Cases n: 1663  . Follow-up: 6.6 to 8.4 years (median) | . Age: 52.2 (7.2) to 62.2 (5.2) years  . Post-menopausal: NR  . Invasive: 100%  . ER+: 59.3% to 72.6% | . Timing: before cancer  . Time to diagnosis: NR  . Dried blood spots, buffy coat, or lymphocytes  . Cell composition: Houseman estimation  . CpGs: n=365,145 *(common to the four studies)* | . Functional normalization (Illumina) and SWAN  . Detection p-value <0.01  . Cross-hybridizing probes: excluded  . SNP probes: NR  . Y chrom: excluded  . X chrom: included | . Beta-values  . Conditional (MCCS, EPIC-Italy, EPIC-IARC) or unconditional (PLCO) logistic regression then fixed-effects meta-analysis  . p<0.05 for average and FDR<0.05 for DMP | *Selection method:* NR  *Variables:*  . Matching variables specific to each study (age)  . Cell-type proportions  . Batch effects (plate or SVA) | *per one SD*  *increase:*  OR=0.94  [0.85-1.05]  I^2^= 0% | *Overall:*  None (0 CpG)  *Within 2 years*  *of blood draw:*  2 CpGs  cg00899463  cg07145930  *(driven by one study)* | . Incomplete adjustment for breast cancer risk factors  . PLCO used the EPIC beadchip |
| Gào X. et al. 2019 [3] | . ESTHER study  (Germany) | . Case-cohort study  . Lag-time: first 2 years (sensitivity analysis)  . Total n: NR (random sub-cohort of n=741)  . Cases n: 128  . Follow-up: NR  *From 2000-2002 to the end of 2014* | . Age: range, 50-74 years  . Post-menopausal: NR  . Invasive: NR  . ER+: NR | . Timing: before cancer  . Time to diagnosis: NR  . Whole blood  . Cell composition: Houseman estimation  . CpGs: n= 3,811  *(promoter regions of 519 genes involved in Oxidative Stress)* | . Functional normalization (Illumina)  . Detection p-value <0.05  . Cross-hybridizing probes: NR  . SNP probes: NR  . Y chrom: NR  . X chrom: excluded | . Beta-values  . Weighted Cox regression then fixed effects meta-analysis  . p<0.05 | *Selection method:* NR  *Variables:*  . Age  . Cell-type proportions  . Batches | NR | 1 CpG  *cg08862778, Tert3 vs Tert1*  HR=0.57  [0.33- 0.97] | . 10/3,811 CpG probes associated with 8-isoprostane levels (P< 7.5×10^−4^) selected for meta-analysis of which 2 CpG replicated (FDR <0.05)  . Incomplete adjustment for breast cancer risk factors  . Same results (NR) after exclusion of 2 first years cases |
| Hofstatter et al. 2019 [4] | . WHI (USA) | . Cohort study  . Lag-time: not considered. Total n: 2107  . Cases n: NR  . Follow-up: NR  (over up to 20+ years) | . Age: NR  . Post-menopausal: NR  . Invasive: 100%  . ER+: NR | . Timing: before cancer  . Time to diagnosis: NR  . Whole blood  . Cell composition: estimated (method NR)  . CpGs: n=NR  *(algorithms of pre-specified 353* CpGs *for Horvath epigenetic clock and 71 CpGs for Hannum epigenetic clock)* | . Preprocessing: NR  . QC: NR  . Cross-hybridizing probes: NR  . SNP probes: NR  . Y chrom: NR  . X chrom: NR | . Type of methylation value: NR  . Cox proportional hazard models  . p<0.05 | *Selection method:* NR  *Variables:*  . Chronologic age  . Observational vs. clinical trial, clinical trial arm  . Race/ethnicity  . Education  . BMI  . Waist-hip ratio  . Smoking  . Alcohol  . Age at menopause  . Age at menarche  . Number of pregnancies  . Age at first birth  . Months breastfed  . Previous mastectomy  . Cell count estimates | *Horvath*  *epigenetic age:*  HR=1.09,  p=6.3x10^-5^  *Hannum clock:*  HR=0.95,  p=0.077 | NR | . Conference abstract only |
| **Table S2. Studies of blood-derived methylation and breast cancer risk (continued)** | | | | | | | | | | |
| **Study reference(s)** | **Population**  **source** | **Design**  Mean (SD), [range] | **Patients and tumors characteristics***  Mean (SD), [range] | **Methylation measurement** | **Preprocessing and statistical modeling** | **Statistical modeling** | | **Reported associations** | | **Comments** |
|  |  |  |  |  |  | **Model(s)** | **Adjustment** | **Average** | **DMP** |  |
| Bermejo J.L. et al. 2019 [5] | . SKKDKFZS (Germany)  . GENICA (Germany) | . Case-control study  . Lag-time: not considered  . Total n: 464  . Cases n: 233  . Follow-up: NA | . Age: median=58.6 years  . Post-menopausal: 72.1%  . Invasive: 98.7%  . ER+: 0% | . Timing: after cancer, before treatment  . Time to diagnosis: NA  . Blood  . Cell composition: adjustment without estimation  . CpGs: n=370,706 | . Preprocessing: Illumina  . Detection p-value <0.01  . Cross-hybridizing probes: NR  . SNP probes: excluded  . Y chrom: NR  . X chrom: NR | . Beta-values  . Factored Spectrally Transformed Linear Mixed Model  . p<0.001 and methylation difference of at least 1% | *Selection method:* NR  *Variables:*  Age | NR | 65 CpGs | . Triple negative breast cancers only  . Controls not drawn from the population that gave rise to cases  . Incomplete adjustment for breast cancer risk factors |
| Li S. et al. 2019 [6] | . AMDTSS  (Australia)  . MCCS  (Australia) | . Cross-sectional study  . Lag-time: NA  . Total n:  AMDTSS : n=436  MCCS : n=591  . Cases n: NA  . Follow-up: NA | . Age:  AMDTSS : 55.6 (8.1)  MCCS : 59.5 (7.3)  . Post-menopausal: AMDTSS : 70%  MCCS: 74%  . Invasive: NA  . ER+: NA | . Timing: before cancer  . Time to diagnosis: NA  . Dried blood spots, buffy coat, or lymphocytes  . Cell-type proportions: Estimated (method NR)  . CpGs: 439,085  *(common to both datasets)* | . Functional normalization (Illumina) and SWAN  . QC: NR  . Cross-hybridizing probes: NR  . SNP probes: NR  . Y chrom: NR  . X chrom: excluded | . M-values  . Linear mixed-effects models then fixed effect meta-analysis  . p<3.4 x 10^−7^ | *Selection method:* NR  *Variables:*  . Age at blood draw  . Difference between age at mammogram and age at blood draw  . BMI  . Smoking status  . Menopausal status  . HRT  . Number of live births  . Cell-type proportions | NR | None (0 CpG) | . Breast mammographic density as outcome |
| Yang Y. et al. 2019 [7] | . FHS (USA)  . WHI (USA)  . BCAC (USA) | . Case-control study (unspecified)  . Lag-time: not considered  . Total n:  FHS : n=1595  WHI : n=883  BCAC: n=228,951  . Cases n:  FHS : n=NA  WHI : n=NA  BCAC: n=122,977  . Follow-up: NR | . Age: NR  . Post-menopausal: NR  . Invasive: NR  . ER+: NR | . Timing: NR  . Time to diagnosis: NR  . White blood cells  . Cell-type proportions: Estimated (SmartSVA method)  . CpGs: 62,938 CpGs (*estimated methylation*) | . Quantile normalization  . QC: NR  . Correlation between predicted and measured methylation <0.01 in FSH, R^2^<0.10 in WHI  . Available SNPs data in BCAC  . Cross-hybridizing probes: NR  . SNP probes: excluded  . Y-chrom: excluded  . X chrom: excluded | . Genetically predicted DNA methylation (beta values used for methylation prediction models)  . Elastic net method for prediction model (FHS), MetaXcan method  . P<7.94×10^-7^ *(Bonferonni)* | *Selection method:* NR  *Variables:*  *.* Age  . Sex  . Cell type composition  . Top ten principal components | NR | 450 CpGs | . FHS and WHI for genetically estimated methylation  . R^2^: squared value of Spearman’s correlation coefficient  . 38/450 CpGs with consistent directions of associations across DNA methylation, gene expression and breast cancer risk |
| Xu Z et al. 2019 [8]  Kresovich J.K. et al. 2019 [9]  O'Brien K.M.et al. 2018 [10] | . Sister study  (USA and  Puerto Rico)  . EPIC-Italy | . Case-cohort and case-control (unspecified) study  . Lag-time: not considered  . Total n:  SS: n= 2878  EPIC-Italy: n= 329  . Cases n:  SS: n=1,616  EPIC-Italy: n=152  . Follow-up: NR  (from 2003-2009 to March 2015) | . Age:  SS: 58 years  EPIC-Italy: NR  . Post-menopausal: NR  . Invasive:  SS: 88%  EPIC-Italy: 100%  . ER+: NR | . Timing: before cancer  . Time to diagnosis: 3.8 [0.1-9.5] years  . Whole blood  . Cell-type proportions: Houseman estimation  . CpGs: 425,500 CpGs for SS (*NR for EPIC-Italy*) | . ENmix /RELIC and RCP method  . <5% low quality data,  . Cross-hybridizing probes: excluded  . SNP probes: excluded  . Y-chrom: excluded  . X chrom: included  . Exclusion of probes with multi-modal distribution | . M-values for DMP (NR for average)  . Kolmogorov-Smirnov nonparametric test for average, Cox proportional hazards model for DMP in SS and logistic regression in EPIC-Italy  . p<0.05 for average, FDR<0.01 and Bonferroni p<10^–7^ for DMP | *Selection method:* NR  *Variables: (DMP)*  . Age  . Cell type proportions  . Top six surrogate variables and experimental plate | . No difference in distribution  . Epigenetic clocks:  Hannum:  HR=1.10  [1.00- 1.21]  Horvath:  HR=1.08  [1.00-1.17]  Levine: HR=1.15  [1.07-1.23] | *All cases:*  806/3452  CpGs  replicated  (FDR<0.01)  *DCIS*  *excluded:*  72/510 CpGs  replicated  (p<10^–7^) | . SS: discovery set; EPIC-Italy: validation set  . Incomplete adjustment for breast cancer risk factors for DMP; no adjustment for average methylation comparison; |
| **Table S2. Studies of blood-derived methylation and breast cancer risk (continued)** | | | | | | | | | | |
| **Study reference(s)** | **Population**  **source** | **Design**  Mean (SD), [range] | **Patients and tumors characteristics***  Mean (SD), [range] | **Methylation measurement** | **Preprocessing and statistical modeling** | **Statistical modeling** | | **Reported associations** | | **Comments** |
|  |  |  |  |  |  | **Model(s)** | **Adjustment** | **Average** | **DMP** |  |
| Campanella G. et al. 2018 [11] | . EPIC-Italy  . EPIC-  Netherlands  . NOWAC  (Norway)  . NSHDS  (Norway) | . Nested case-control studies  . Lag-time: 1 year  . Total n: 565  . Cases n: 283  . Follow-up: NR | . Age: NR  . Post-menopausal: 100%  . Invasive: NR  . ER+: NR | . Timing: before cancer  . Time to diagnosis: NR  . Peripheral blood leucocytes  . Cell-type proportions: not considered  . CpGs: 40 adiposity-related CpG *(p<1.06×10^-7^)* | . Preprocessing: NR  . Detection p-value <0.05  . Cross-hybridizing probes: excluded  . SNP probes: NR  . Y chrom: excluded  . X chrom: excluded | . Beta-values  . Generalised linear model for beta-distribution / linear mixed model for paired samples then fixed-effect meta-analysis for association with adiposity; logistic regression model for association with breast cancer risk  . p<1.06×10^−7^ for adiposity; p<1,67x10^-3^ for breast cancer risk | *Selection method:* NR  *Variables:*  . Age  . Alcohol  . Contraceptive use  . HRT  . Smoking status  . Physical activity  . With and without adjustment for BMI and WHR  . Microarray position for association with adiposity  . Cell type proportions (sensitivity analysis) | NR | None (0 CpG) | . Bonferroni-corrected significance level assuming n=30 (principal components needed to explain more than 95% of the variance) independent tests for breast cancer risk  . Incomplete adjustment for breast cancer risk factors |
| Joo J.E. et al. 2018 [12] | . MCBCF (USA  and Australia  . MCCS  (Australia) | . Nested case-control studies  . Lag-time: not considered  . Total n:  MCBCF: n=210  MCCS: n=866  . Cases n:  MCBCF: n=87  MCCS: n=433  . Follow-up:  MCBCF: NR  MCCS: median=9.5  IQR 5.0 to 13.1 years | . Age: NR  . Post-menopausal: NR  . Invasive:  MCBCF: NR  MCCS: 100%  . ER+: NR | . Timing:  MCBCF: NR  MCCS: before cancer  . Time to diagnosis: NR  . Whole peripheral blood, dried blood spot, buffy coat, or lymphocyte  . Cell-type proportions: Houseman estimation  . CpGs: 1,000/365,169 most Mendelian methylation marks | . Functional normalization (Illumina) and SWAN  . Detection p-value <0.05  . Cross-hybridizing probes: NR  . SNP probes: excluded  . Y chrom: excluded  . X chrom: excluded | . M-values  . Mixture model then Cox proportional hazards survival analysis for associations between breast cancer and the carrier probabilities for the 1000 most Mendelian methylation marks (MCBCF)  . Conditional logistic regression models (MCCS)  . p<5x10^-5^  for MCBCF;p<0.05 for MCCS | *Selection method:* NR  *Variables: (MCCS only)*  . Age  . BMI  . Smoking  . Alcohol drinking  . Time between blood collection and cancer  . Sample type  *Sensitivity analysis*  *(MCCS only)*  . Age at menarche  . Menopausal status  . Number of live births  . HRT  . Blood cell composition | NR | 4/24 CpGs in MCBCF replicated in MCCS:  . cg18584561  OR=1.18  [1.03-1.36]  . cg01741999  OR=1.26  [1.03-1.54]  . cg03916490  OR=0.83  [0.72-0.96]  . cg27639199  OR=1.19  [1.03-1.36]  *(OR per 1SD)* | . Complete adjustment for breast cancer risk factors only in MCCS |
| Scott C.M. et al. 2018 [13] | . ABCFS  (Australia) | . Nested case-control study  . Lag-time: not considered  . Total n: 90  . Cases n: 60  . Follow-up: NR | . Age: <40 years old  . Post-menopausal: NR  . Invasive: NR  . ER+: NR | . Timing: after cancer  (8 months after cancer)  . Time to diagnosis: NA  . Dried blood spots  . Cell-type proportions: Houseman estimation  . CpGs: 477,380 | . Functional normalization (Illumina) and SWAN  . Detection p-value <0.05  . Cross-hybridizing probes: NR  . SNP probes: NR  . Y chrom: NR  . X chrom: included | . Type of methylation value: NR  . Linear regression (limma)  . FDR<0.01 | *Selection method:* NR  *Variables:*  . Age (matching) | NR | None (0 CpG) | . Incomplete adjustment for breast cancer risk factors |
| Durso DF et al. 2017 [14] | . EPIC-Italy,  Turin | . Cohort study and nested case-control study  . Lag-time: not considered  . Total n: 573  . Cases n: 233  . Follow-up: NR | . Age: 52.4 (7.4)  . Post-menopausal: NR  . Invasive: NR  . ER+: NR | . Timing: before cancer  . Time to diagnosis: NR  . Buffy coats  . Cell-type proportions: Houseman estimation  . CpGs: NR  *(algorithm based on 353* CpGs *for Horvath, 71 CpGs for Hannum, 3 CpGs for* Weidner *epigenetic clocks and 2 CpGs specific: cg16867657 (ELOV2) and cg06639320 (FHL2)* | . Preprocessing: NR  . Detection p-value: NR  . Cross-hybridizing probes: NR  . SNP probes: NR  . Y chrom: NR  . X chrom: NR | . Beta-values  . Mann-Whitney-Wilcoxon test and Log-rank test (Kaplan-Meier)  . p<0.05 | *Selection method:* NR  *Variables:*  . Blood cell counts  *(sensitivity analysis)* | None | 1 CpG  . cg16867657  *(Mann-*  *Whitney-*  *Wilcoxon test*  *p-value =0.04,*  *lost when*  *correction for*  *blood cell*  *counts)* | . No adjustment for breast cancer risk factors |
| **Table S2. Studies of blood-derived methylation and breast cancer risk (continued)** | | | | | | | | | | |
| **Study reference(s)** | **Population**  **source** | **Design**  Mean (SD), [range] | **Patients and tumors characteristics***  Mean (SD), [range] | **Methylation measurement** | **Preprocessing and statistical modeling** | **Statistical modeling** | | **Reported associations** | | **Comments** |
|  |  |  |  |  |  | **Model(s)** | **Adjustment** | **Average** | **DMP** |  |
| Ambatipudi S. et al. 2017 [15] | . EPIC (Europe) | . Nested case-control study  . Lag-time: not considered  . Total n: 960  . Cases n: 480  . Follow-up: NR | . Age: 52.3 (8.97)  . Post-menopausal: 59%  . Invasive: 100%  . ER+: 83% | . Timing: before cancer  . Time to diagnosis: NR  . Buffy coats  . Cell-type proportions: Houseman estimation  . CpGs: 421,583;  Horvath epigenetic clock (based on 353 CpGs) | . Beta-mixture quantile normalisation (BMIQ)  . Detection p-value<0.05  . Cross-hybridizing probes: Excluded  . SNP probes: Excluded  . Y chrom: NR  . X chrom: NR | . Beta-values  . Conditional logistic regression model for average methylation, unconditional logistic regression for Horvath clock  . p<0.05 | *Selection method:* NR  *Variables:*  Average and DMP:  . Technical batch effects  . Alcohol consumption  . BMI  IEAA:  . Age  . Alcohol consumption  . Full term pregnancy  . BMI  . Level of education  . Age at menarche  . Cambridge physical activity index  . Cell types estimates | . No difference globally for average methylation;  . CpG island sites:  OR=1.20  [1.03-1.40]  . IEAA:  OR=1.04  [1.01-1.08]  *(OR per 1SD)* | None (0 CpG) | . Intrinsic Epigenetic Age Acceleration (IEAA): residues of regression of Horvath epigenetic clock on age and corrected for cell types estimates  . Association with Horvath epigenetic clock driven by postmenopausal women  . Incomplete adjustment for breast cancer risk factors for DMP analysis |
| Dugué P.A et al. 2017 [16]  Severi G. et al. 2014 [17] | . MCCS  (Australia) | . Nested case-control study  . Lag-time: not considered  . Total n: 840  . Cases n: 420  . Follow-up: NR | . Age: 64.0 (8.0), [44-83]  . Post-menopausal: NR  . Invasive: 100%  . ER+: 71% | . Timing: before cancer  . Time to diagnosis: NR  . Dried blood spot, buffy coats or lymphocytes  . Cell-type proportions: Houseman estimation  . CpGs: 481,273 for average, 7 CpGs for DMP *(identified and replicated or validated in Tang Q.Q. et al.)* | . Normalization to internal controls (Illumina) and SWAN  . Detection p-value<0.01  . Cross-hybridizing probes: NR  . SNP probes: NR  . Y chrom: NR  . X chrom: NR | . Beta values for average, M-values for DMP  . Linear mixed effect models and Conditional logistic regression for average, conditional logistic regression for DMP  . p<0.05 | *Selection method:* NR  *Variables: (matching)*  Average:  . Age  . Type of biospecimen  . Country of origin  DMP:  . Age  . Ethnicity  . Cell counts | . Globally:  OR=0.69  [0.50, 0.95]  . <5 years from diagnosis:  OR=0.49  [0.28,0.86]  . Far from islands  OR=0.63  [0.48, 0.82]  . Functional promoters  OR=1.82  [1.20, 2.75]  . Outside promoters  OR=0.63  [0.46, 0.85]  *(OR for 1 SD)* | None | . Average analyses adjusted for breast cancer risk factors, comorbidity and cell counts in sensitivity analysis; DMP analyses adjusted for smoking status, BMI, alcohol drinking and cell counts in sensitivity analysis  . Incomplete adjustment for DMP analyses  . Global average results driven by the group <5 years between blood collection and diagnosis  . No association for islands and shores |
| Tang Q.Q.  et al. 2016 [18] | . GC-HBOC  (Germany) | . Case-control study (hospital controls)  . Lag-time: not considered  . Total n: 96  . Cases n: 48  . Follow-up: NR | . Age: 47.7 (7.2)  . Post-menopausal: 31%  . Invasive: NR  . ER+: 85% | . Timing: after cancer, before treatment  . Time to diagnosis: NR  . Whole blood  . Cell-type proportions: Houseman estimation  . CpGs: 392,370 | . Functional normalization (Illumina) and quantile normalization  . Detection p-value<0.01  . Cross-hybridizing probes: NR  . SNP probes: NR  . Y chrom: NR  . X chrom: NR | . Beta-values  . NR for average, Beta-regression models with a logistic link for DMP  . p<0.05 for average, FDR<0.005 for DMP | *Selection method:* NR  *Variables:*  Average: NR  DMP:  . Age  . Batch  . Cell counts | Trend towards marginally lower DNA methylation cases  (mean β=52.33% vs 52.41%, p= 0.089) | 20/136 CpGs with p≤1.4x10^-06^, Δβ> 4% and not an intergenic site of which 7 reported:  cg06418238^†^  cg00736299^†^  cg27466532^†^  cg06526620  cg21932542  cg22941668  cg22233512 | . Incomplete adjustment for breast cancer risk factors  . n=7 reported CpGs fulfilling requirements of validation by MassARRAY Epityper (all hypomethylated in cases)  . n=3 CpGs validated by MassARRAY EpiTyper (^†^) |
| **Table S2. Studies of blood-derived methylation and breast cancer risk (continued)** | | | | | | | | | | |
| **Study reference(s)** | **Population**  **source** | **Design**  Mean (SD), [range] | **Patients and tumors characteristics***  Mean (SD), [range] | **Methylation measurement** | **Preprocessing and statistical modeling** | **Statistical modeling** | | **Reported associations** | | **Comments** |
|  |  |  |  |  |  | **Model(s)** | **Adjustment** | **Average** | **DMP** |  |
| Van  Veldhoven K. et al. 2015 [19]  Cordero F. et al. 2015 [20] | . EPIC-Italy  . NOWAC  (Norway)  . Generations  Study (UK) | . Nested case-control studies  . Lag-time: not considered  . Total n:  EPIC-Italy: n=324  NOWAC: n=338  GS: n=1096  . Cases n:  EPIC-Italy: n=166  NOWAC: n=192  GS: n=548  . Follow-up:  EPIC-Italy:  mean=106.8 months  range [0.53-188.8]  NOWAC: NR  GS: NR | . Age:  EPIC-Italy: 52.1 (8.08)  NOWAC: 55.4 [47.2-63.3]  GS: 52.0 [24.0-82.0]  . Post-menopausal:  EPIC-Italy: 62%  NOWAC: 78.6%  GS: NR  . Invasive:  EPIC-Italy: NR  NOWAC: NR  GS: 78%  . ER+:  EPIC-Italy: 34.6%  NOWAC: 77.4%  GS: 66% | . Timing: before cancer  . Time to diagnosis:  2.1 to 5.3 [0-15.7] years  . Buffy coats  . Cell-type proportions: Houseman estimation  . CpGs: 407,455  (common to EPIC-Italy and NOWAC) | . Beta-mixture quantile normalisation (BMIQ)  . Detection p-value<0.05  . Cross-hybridizing probes: NR  . SNP probes: NR  . Y chrom: NR  . X chrom: NR | . Beta-values  . Conditional logistic regression, robust logistic regression and B-spline regression model for average, Generalised linear model for beta-distribution and Poisson GLM for DMP  . p<0.05 for average, p<1.2x 10^−7^ (Bonferroni) for DMP | *Selection method:* NR  *Variables:*  Average:  . Age  . Cell counts  DMP:  . Age  . Technical confounders  . Cell counts *(sensitivity analysis)* | . EPIC-Italy:  OR=0.71  [0.61–0.84]  . NOWAC:  OR=1.03  [0.82–1.30]  . GS:  0.2% mean  hypomethylated  in cases; in  gene body  (66.1% vs  66.5%); no  difference in  islands.  *(OR for 1 SD)* | 26 CpGs in EPIC-Italy ;  None in NOWAC | . Incomplete adjustment for breast cancer risk factors  *(no association between epigenome-wide-methylation and risk factors)*  . Whole genome sequencing in Generations study; no DMP analysis in Generations Study |
| Shenker N.S. et al. 2013 [21] | . EPIC-Turin, Torino sub-cohort (Italy) | . Nested case-control study  . Lag-time: 2 years  . Total n: 184  . Cases n: 92  . Follow-up: NR | . Age: median=51 [34-65]  . Post-menopausal: NR  . Invasive: NR  . ER+: 45% | . Timing: before cancer  . Time to diagnosis:  31% < 5 years  34% > 10 years  . Buffy coats or blood cell fractions  . Cell-type proportions: Not considered  . CpGs: 9/484,804 associated with smoking | . Normalization to internal controls (Illumina) and quantile normalization  . Detection p-value<0.05  . Cross-hybridizing probes: NR  . SNP probes: not excluded  . Y chrom: NR  . X chrom: NR | . Beta-values  . Attributable risk (model NR)  . p<0.05 | *Selection method:* NR  *Variables:*  . Age  . Seasonality of blood collection  . Duration of follow-up  . Smoking  . ER status | NR | 2 CpGs  cg21566642  cg01940273 | . Incomplete adjustment for breast cancer risk factors |
| SD: standard deviation; n: number; NR: not reported; NA: not applicable; SNP: single nucleotide polymorphism; DMP: differentially methylated positions; CpG: Cytosie-phophate-Guanine; BMI: body mass index; WHR: Waist-hip ratio; *HRT: hormone replacement therapy;* IQR: interquartile range; QC: quality control; chrom: chromosome; FDR: false discovery rate; GLM: Generalised linear model; SWAN: subset-quantile within array normalization; RCP: Regression on Correlated Probes  EPIC: European Prospective Investigation into Cancer and Nutrition ; IARC: International Agency for Research on Cancer; MCCS: Melbourne Collaborative Cohort Study; PLCO: Prostate, Lung, Colorectal, and Ovarian Cancer Screening Trial; WHI: Women’s health initiative; AMDTSS: AustralianMammographic Density Twins and Sisters Study; FHS: Framingham Heart Study; BCAC: Breast Cancer Association Consortium; SS: Sister Study; NOWAC: Norwegian Women and Cancer Study; NSHDS : Northern Sweden Health and Disease Study; MCBCF: Multiple-case breast cancer families; ABCFS: Australian Breast Cancer Family Study; GC-HBOC: German Consortium for Hereditary Breast and Ovarian Cancer; GS: Generations Study; SKKDKFZS: Stadtisches Klinikum Karlsruhe Deutsches Krebsforschungszentrum; GENICA: Gene Environment Interaction and Breast Cancer in Germany; | | | | | | | | | | |

| **Table S3. Studies of breast tissue methylation and breast cancer risk** | | | | | | | | | | |
| --- | --- | --- | --- | --- | --- | --- | --- | --- | --- | --- |
| **Study reference(s)** | **Population**  **source** | **Design**  Mean (SD), [range] | **Patients and tumors characteristics***  Mean (SD), [range] | **Methylation measurement** | **Preprocessing and statistical modeling** | **Statistical modeling** | | **Reported associations** | | **Comments** |
|  |  |  |  |  |  | **Model(s)** | **Adjustment** | **Average** | **DMP** |  |
| Xiao B. et al. 2018 [22] | NR  *(TCGA data)* | . Design: NR  . Lag-time: NA  . Total n: 231  . Cases n: 210  . Follow-up: NA | . Age:  Tumor tissue: 60.7 (13.2)  Normal tissue: 56.4 (14.6)  . Post-menopausal: NR  . Invasive: 95% *(tumor tissue patients)*  . ER+: 97% *(tumor tissue patients)* | . Timing: after cancer  . Tumor tissue vs normal tissue *(from different breast cancer patients, sample preservation not reported)*  . Cell-type proportions: not verified, not considered  . CpGs: NR | . Preprocessing: NR  . Detection p-value: NR  . Cross-hybridizing probes: NR  . SNP probes: NR  . Y chrom: NR  . X chrom: NR | . Beta-values  . Wilcoxon rank sum test  . FDR and p-values <0.0001 | *Selection method:* NR  *Variables:* None | NR | 550 CpGs  *(tumor vs normal tissue)* | . No adjustment for breast cancer risk or prognostic factors |
| Hofstatter E.W. et al. 2018 [23] | . Yale Breast  Center  .Susan G. Komen  Tissue Bank  .Yale New Haven  Hospital | . Cross-sectional  . Lag-time: NA  . Total n: 96  . Cases n: 35  . Follow-up: NA | . Age: 49.7,  95% CI (46.3–53.0)  . Post-menopausal: 33%  . Invasive: NR  . ER+: 97.5% | . Timing: after cancer, before treatment  . Normal breast tissue *(fixed in formalin and frozen)*  . Cell-type proportions: not verified, not considered  . CpGs: Horvath epigenetic clock (based on 353 CpGs) | . Noob normalization  . Detection p-value: NR  . Cross-hybridizing probes: NR  . SNP probes: NR  . Y chrom: NR  . X chrom: NR | . Beta-values  . Non-parametric tests  . p<0.05 | *Selection method:* NR  *Variables:*  . Age  . Smoking status  *(matching)* | . Higher mean of DNAmAge in  cases (average  difference of  3.98 years, p=  0.003) | NR | . Controls not drawn from the population that gave rise to cases  . Incomplete adjustment for breast cancer risk or prognostic factors |
| Ambrosone C.B. et al. 2014 [24] | . Pathology  Resource  Network at  Roswell  Park Cancer  Institute | . Cross-sectional  . Lag-time: NA  . Total n: 262  . Cases n: 138  . Follow-up: NA | . Age: 29.0 % <49,  35.5% [50-68],  35,5% >69 years old  . Post-menopausal: NR  . Invasive: 99%  . ER+: 63% | . Timing: after cancer  . Tumor tissue of cases vs normal tissue of non- cases *(snap frozen)*  . Cell-type proportions: not verified, not considered  . CpGs: 276,108 | . SWAN  . Detection p-value <0.05  . Cross-hybridizing probes: excluded  . SNP probes: excluded  . Y chrom: NR  . X chrom: NR | . Beta-values  . Welch’s t-test for average, Wilcoxon rank-sum test for DMP  . p<0.05 for average, FDR <0.05 and \|delta β\|=0.17 for DMP | *Selection method:* NR  *Variables:* None | . Higher in cancer samples globally and in islands and shores  . Lower in cancer samples in shelves and “open-see” | 2,761 CpGs  *(list not*  *reported)* | . No adjustment for breast cancer risk or prognostic factors |
| SD: standard deviation; n: number; NR: not reported; NA: not applicable; SNP: single nucleotide polymorphism; DMP: differentially methylated positions; CpG: Cytosie-phophate-Guanine; CI: confidence interval;  BMI: body mass index; WHR: Waist-hip ratio; *HRT: hormone replacement therapy;* IQR: interquartile range; QC: quality control; chrom: chromosome; FDR: false discovery rate; GLM: Generalised linear model; SWAN: subset-quantile within array normalization; SWAN: subset-quantile within array normalization | | | | | | | | | | |

**References**

1. Johansson A, Palli D, Masala G, et al. Epigenome-wide association study for lifetime estrogen exposure identifies an epigenetic signature associated with breast cancer risk. Clin Epigenetics 2019;11(1):66.

2. Bodelon C, Ambatipudi S, Dugue PA, et al. Blood DNA methylation and breast cancer risk: a meta-analysis of four prospective cohort studies. Breast Cancer Res 2019;21(1):62.

3. Gao X, Zhang Y, Burwinkel B, et al. The associations of DNA methylation alterations in oxidative stress-related genes with cancer incidence and mortality outcomes: a population-based cohort study. Clin Epigenetics 2019;11(1):14.

4. Hofstatter EW, Levine M, Hatzis C, et al. Age-related methylation signals of breast cancer risk in blood. Cancer Research 2019;79(4).

5. Bermejo JL, Huang G, Manoochehri M, et al. Long intergenic noncoding RNA 299 methylation in peripheral blood is a biomarker for triple-negative breast cancer. Epigenomics 2019;11(1):81-93.

6. Li S, Dugue PA, Baglietto L, et al. Genome-wide association study of peripheral blood DNA methylation and conventional mammographic density measures. Int J Cancer 2019;145(7):1768-1773.

7. Yang Y, Wu L, Shu XO, et al. Genetically predicted levels of DNA methylation biomarkers and breast cancer risk: data from 228,951 women of European descent. J Natl Cancer Inst 2019; 10.1093/jnci/djz109.

8. Xu Z, Sandler DP, Taylor JA. Blood DNA methylation and breast cancer: A prospective case-cohort analysis in the Sister Study. J Natl Cancer Inst 2019; 10.1093/jnci/djz065.

9. Kresovich JK, Xu Z, O'Brien KM, et al. Methylation-Based Biological Age and Breast Cancer Risk. Journal of the National Cancer Institute 2019;111(10):1051-1058.

10. O'Brien KM, Sandler DP, Xu Z, et al. Vitamin D, DNA methylation, and breast cancer. Breast cancer research : BCR 2018;20(1):70-70.

11. Campanella G, Gunter MJ, Polidoro S, et al. Epigenome-wide association study of adiposity and future risk of obesity-related diseases. Int J Obes (Lond) 2018;42(12):2022-2035.

12. Joo JE, Dowty JG, Milne RL, et al. Heritable DNA methylation marks associated with susceptibility to breast cancer. Nat Commun 2018;9(1):867.

13. Scott CM, Wong EM, Joo JE, et al. Genome-wide DNA methylation assessment of 'BRCA1-like' early-onset breast cancer: Data from the Australian Breast Cancer Family Registry. Exp Mol Pathol 2018;105(3):404-410.

14. Durso DF, Bacalini MG, Sala C, et al. Acceleration of leukocytes' epigenetic age as an early tumor and sex-specific marker of breast and colorectal cancer. Oncotarget 2017;8(14):23237-23245.

15. Ambatipudi S, Horvath S, Perrier F, et al. DNA methylome analysis identifies accelerated epigenetic ageing associated with postmenopausal breast cancer susceptibility. Eur J Cancer 2017;75:299-307.

16. Dugue PA, Milne RL, Southey MC. A prospective study of peripheral blood DNA methylation at RPTOR, MGRN1 and RAPSN and risk of breast cancer. Breast Cancer Res Treat 2017;161(1):181-183.

17. Severi G, Southey MC, English DR, et al. Epigenome-wide methylation in DNA from peripheral blood as a marker of risk for breast cancer. Breast Cancer Res Treat 2014;148(3):665-73.

18. Tang Q, Holland-Letz T, Slynko A, et al. DNA methylation array analysis identifies breast cancer associated RPTOR, MGRN1 and RAPSN hypomethylation in peripheral blood DNA. Oncotarget 2016;7(39):64191-64202.

19. van Veldhoven K, Polidoro S, Baglietto L, et al. Epigenome-wide association study reveals decreased average methylation levels years before breast cancer diagnosis. Clin Epigenetics 2015;7:67.

20. Cordero F, Ferrero G, Polidoro S, et al. Differentially methylated microRNAs in prediagnostic samples of subjects who developed breast cancer in the European Prospective Investigation into Nutrition and Cancer (EPIC-Italy) cohort. Carcinogenesis 2015;36(10):1144-1153.

21. Shenker NS, Polidoro S, van Veldhoven K, et al. Epigenome-wide association study in the European Prospective Investigation into Cancer and Nutrition (EPIC-Turin) identifies novel genetic loci associated with smoking. Hum Mol Genet 2013;22(5):843-51.

22. Xiao B, Chen L, Ke Y, et al. Identification of methylation sites and signature genes with prognostic value for luminal breast cancer. BMC Cancer 2018;18(1):405.

23. Hofstatter EW, Horvath S, Dalela D, et al. Increased epigenetic age in normal breast tissue from luminal breast cancer patients. Clin Epigenetics 2018;10(1):112.

24. Ambrosone CB, Young AC, Sucheston LE, et al. Genome-wide methylation patterns provide insight into differences in breast tumor biology between American women of African and European ancestry. Oncotarget 2014;5(1):237-48.

| **Correspondence between reference citation order in supplemental tables and manuscript main text** | | |
| --- | --- | --- |
| **Reference** | **Citation order in supplemental tables** | **Order In manuscript main text** |
| Johansson A. et al. 2019 | [1] | [24] |
| Bodelon C. et al. 2019 | [2] | [25] |
| Gào X. et al. 2019 | [3] | [26] |
| Hofstatter et al. 2019 | [4] | [27] |
| Bermejo J.L. et al. 2019 | [5] | [28] |
| Li S. et al. 2019 | [6] | [29] |
| Yang Y. et al. 2019 | [7] | [30] |
| Xu Z et al. 2019  Kresovich J.K. et al. 2019  O'Brien K.M.et al. 2018 | [8], [9], [10] | [31] |
| Campanella G. et al. 2018 | [11] | [32] |
| Joo J.E. et al. 2018 | [12] | [33] |
| Scott C.M. et al. 2018 | [13] | [34] |
| Durso DF et al. 2017 | [14] | [35] |
| Ambatipudi S. et al. 2017 | [15] | [36] |
| Dugué P.A et al. 2017  Severi G. et al. 2014 | [16], [17] | [37], [44] |
| Tang Q.Q. et al. 2016 | [18] | [38] |
| Van Veldhoven K. et al. 2015  Cordero F. et al. 2015 | [19], [20] | [39] |
| Shenker N.S. et al. 2013 | [21] | [40] |
| Xiao B. et al. 2018 | [22] | [41] |
| Hofstatter E.W. et al. 2018 | [23] | [42] |
| Ambrosone C.B. et al. 2014 | [24] | [43] |
